# Acetylation of Microcrystalline Cellulose by Transesterification in AmimCl/DMSO Cosolvent System

**DOI:** 10.3390/molecules22091419

**Published:** 2017-08-27

**Authors:** Huihui Wang, Xiaoxiang Wen, Xueqin Zhang, Chuanfu Liu

**Affiliations:** State Key Laboratory of Pulp and Paper Engineering, South China University of Technology, Guangzhou 510640, China; wang.huihui@mail.scut.edu.cn (H.W.); 15902078732@163.com (X.W.); xueqin0228@gmail.com (X.Z.)

**Keywords:** cellulose acetate, ionic liquid, cosolvent, transesterification, DMSO

## Abstract

Recently, IL/cosolvent systems have generated a lot of interest as cellulose-dissolving solvents and reaction media for various kinds of cellulose modification. In the present study, both 1-allyl-3-methylimidazolium chloride (AmimCl)/dimethyl sulfoxide (DMSO) and AmimCl/*N*,*N*-dimethylformamide (DMF) systems were employed to synthesize cellulose acetate by transesterification. Microcrystalline cellulose, 1,8-diazabicyclo[5.4.0]undec-7-ene and isopropenyl acetate were chosen as the raw material, catalyst and acetylation reagent, respectively. The results revealed that DMSO was a suitable cosolvent for the transesterification in the homogeneous solution. Moreover, DMSO had a positive effect on the reaction as the cosolvent under the given conditions and the degree of the substitution of cellulose acetate could be significantly enhanced through increasing the molar ratio of DMSO. The synthesized products were characterized by Fourier transform infrared (FT-IR) spectroscopy, ^1^H and ^13^C nuclear magnetic resonance spectroscopy (^1^H-NMR and ^13^C-NMR), correlation spectroscopy (COSY), heteronuclear single quantum correlation (HSQC) spectroscopy, and X-ray diffraction (XRD) to confirm the chemical and physical structure of the cellulose acetate generated. The thermal properties were also evaluated using thermogravimetric analysis (TGA)/derivative thermogravimetry (DTG).

## 1. Introduction

In the last few decades, cellulose, the most abundant biopolymer, has been broadly studied and applied in many different fields due to advantages such as renewability, biodegradability, and biocompatibility. However, there are some limits in the application of cellulose because it does not dissolve in water or common organic solvents and is difficult to mold by compression and extrusion due to the lack of thermoplasticity. Therefore, chemical modification is a vital method for the application of cellulose.

Cellulose acetate (CA), one of the most commonly used cellulose esters, has been commercially produced for many decades. Due to its great processibility, it could be used to prepare filter membrane, separation membrane, coating materials and so on. Although CA can be achieved in heterogeneous and homogeneous system, CA obtained from homogeneous systems has more uniform structure and degree of substitution (DS) [1]. Recently, it has been reported that several solvents, including dimethyl sulfoxide/tetrabutylammonium fluoride (DMSO/TBAF) [2], *N*,*N*-dimethylacetamide/lithium chloride (DMA_C_/LiCl) [3], *N*-methylmorpholine-*N*-oxide (NMMO) [4] and NaOH/urea [5] could dissolve cellulose. However, these solvent systems are either toxic or applied in extreme conditions.

Fortunately, a novel promising set of cellulose solvents called ionic liquids (ILs), made up of cations and anions, has been developed. Due to their extremely low vapor pressure, ILs would not discharge volatile organic compounds and could be easily separated from volatile components by simple evaporation allowing for recycle processes. The lack of fugitive emissions and ease of recycling makes these suitable “green” solvents for cellulose dissolution and processing. In addition, some types of IL can dissolve large amounts of cellulose-up to 25 wt % [6]. Many attempts have been made to modify cellulose in ILs, often with great success [7,8]. Although ILs have a lot of fascinating properties, the dissolution of cellulose in IL can be time consuming [9] and the high viscosity of the obtained solutions limits the efficiency of cellulose modification. Recently, IL/cosolvent systems have generated a lot of interest [10,11,12]. The addition of cosolvents to the IL can significantly reduce the viscosity, which can accelerate mass transport processes and increase the dissolution rate of cellulose [13]. It was reported that polar aprotic solvents like DMSO, *N*,*N*-dimethylformamide (DMF), and 1,3-dimethyl-2-imidazolidinone (DMI) can act as cosolvents [14]. Although, there have been previous reports on the transesterification of cellulose catalyzed by IL, these processes nonetheless required a great deal of time to dissolve and modify cellulose [15]. As reported in the literature [16], ionic liquid 1-allyl-3-methylimidazolium chloride (AmimCl) could rapidly dissolve cellulose, and the viscosity of the obtained solution was low [17]. To shorten the dissolution and transesterification time, both AmimCl/DMSO and AmimCl/DMF were selected as the reaction media to synthesize CA by transesterification in this study. 1,8-Diazabicyclo[5.4.0]undec-7-ene (DBU) and isopropenyl acetate (IPA) were chosen as catalyst and acetylation reagent, respectively. The products were characterized using Fourier transform infrared (FT-IR) spectroscopy, ^1^H and ^13^C nuclear magnetic resonance spectroscopy (^1^H-NMR and ^13^C-NMR), ^1^H-^1^H correlation spectroscopy (COSY), ^1^H-^13^C heteronuclear single quantum correlation spectroscopy (HSQC), thermogravimetric analysis (TGA)/derivative thermogravimetry (DTG), and X-ray diffraction (XRD). 

## 2. Results and Discussion

### 2.1. Transesterification of Microcrystalline Cellulose (MCC)

As can be seen from Table 1, the transesterification reaction was successfully performed. To investigate the effect of the temperature on the DS, different temperatures were employed for the reaction. It was observed that DS of the CA samples increased when elevating the temperature in different solvent systems. For example, the DS of CA-1, CA-2, CA-3, CA-4 and CA-5 increased from 1.82 at 80 °C to 1.97 at 90 °C, 2.16 at 100 °C, 2.31 at 110 °C and 2.38 at 120 °C, respectively. This increase was probably due to the more flexible cellulose chains and the more active catalyst (DBU) at higher temperature.

The results in Table 1 indicated that the DS of the CA samples produced in AmimCl/DMF cosolvent system was relatively lower under the same conditions, compared with that in neat IL. This implied that, although the addition of DMF could decrease the viscosity and facilitate mixing, it hardly improves the transesterification. On the contrary, the DS of the CA samples synthesized in AmimCl/DMSO was marginally higher than that in neat IL, suggesting that DMSO as the cosolovent had a greater effect on the reaction under the given conditions. The results were similar to the previous literature [18].

To further elucidate the effect of the DMSO on the reaction, different molar ratio of IL and DMSO was investigated. The DS of the sample was improved when increasing the molar ratio of DMSO. This improvement was probably due to the following aspects: the DMSO reduced the viscosity of IL solution, which increased the mass transfer and heat transfer rate; secondly, the interaction between DMSO and the imidazolium cation weakened the interactive force of IL and cellulose hydroxyl group [15], which was helpful to reduce the steric hindrance of the large cations and make the cellulose hydroxyl group more accessible [19]; last but not the least, the synthesized CA could be quickly dissolved in DMSO, avoiding being hydrolyzed after transesterification [20,21]. It was interesting that the total DS could be controlled by adjusting the molar ratio of IL and DMSO. Moreover, the DS of CA-16 was high: up to 2.75 with the molar ratio IL_0.23_/DMSO_0.77_. Herein, the IL acted more like an electrolyte and the cosolvent system was composed essentially of inexpensive DMSO. These results showed cellulose triacetate could be successfully obtained by transesterification in AmimCl/DMSO as a decreased cost homogeneous system, while it could not be achieved in neat AmimCl under the same conditions.

The solubility of the CA samples produced by transesterification in IL/cosolvent system was also examined in organic solvent, including DMSO, DMF and acetone, and found to vary. CA samples (10 g) were added into 0.5 mL of solvent and stirred for 12 h, and the results are recorded in Table 1. The CA samples were insoluble, but swollen, in DMF. As expected, the CA samples dissolved readily in DMSO. However, the solubility in acetone greatly depends on the total DS value. The CA samples with DS more than 2.31 were readily dissolved in acetone, making it possible to produce CA membranes from this volatile solvent. Similar results were also reported recently [22].

### 2.2. FT-IR Spectra

**Figure 1:**
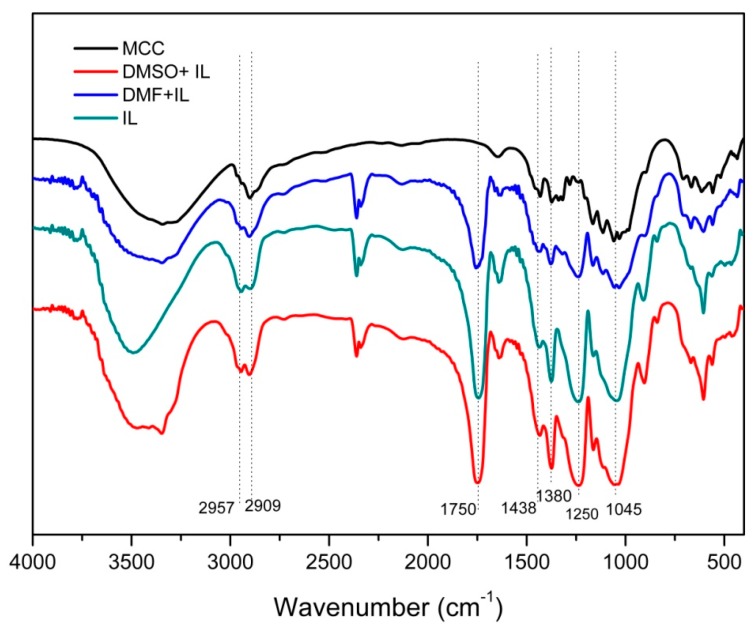
FT-IR spectra of MCC and the prepared CA-5, CA-8 and CA-11 by DBU-catalyzed transesterification.

The FT-IR spectra of the CA samples reacted in different cosolvents are illustrated in Figure 1. Compared with the unmodified MCC, there were several new signals in the spectra of CA-5, CA-8 and CA-11. The band around 1750 cm^−1^ relates to C=O stretching and the peak at 1438 cm^−1^ is associated with the scissoring of the methylene group. The peaks at 1380 cm^−1^ and 1250 cm^−1^ are attributed to the C–H bending and symmetric stretching of C–O in the ester, respectively, while the signal at 1045 cm^−1^ is attributed to the asymmetric stretching of the C–O–C glycosidic bond from the pyranose ring. The peaks at 2909 cm^−1^ and 2957 cm^−1^ originate from asymmetric stretching of the alpha-saturated methyl groups and the methylene groups, respectively [23]. The presence of these absorbances revealed that the CA was successfully synthesized by DBU-catalyzed transesterification in different cosolvent systems.

### 2.3. NMR Analysis

The chemical structure of the synthesized CA sample was further investigated using 1D and 2D NMR. In order to remove the interference of water, a drop of trifluoroacetic acid-*d*_6_ was added. The signals from 1.60 to 2.30 ppm (Figure S1) are attributed to the protons of the methyl group, indicating that the acetylation of cellulose was successfully achieved in the cosolvent system, and the signals between 3.00 and 5.20 ppm arise from to the protons of anhydroglucose units [24]. 

The COSY spectrum reveals the directly coupled ^1^H-^1^H spin couplings, and the HSQC spectrum of CA provides the directly coupled ^1^H-^13^C spin coupling. The correlations in the COSY spectrum of CA with DS of 2.75 (Figure S2A) at δ_H_/δ_H_ 4.69/4.69, 4.54/4.54, 5.09/5.09, 3.68/3.68, 3.84/3.84, 4.01/4.01 and 4.24/4.24 ppm can be assigned to H_1_/H_1_, H_2_/H_2_, H_3_/H_3_, H_4_/H_4_, H_5_/H_5_, H_6`_/H_6`_, and H_6_/H_6_ of the glucose ring. The cross correlations at δ_H_/δ_H_ 5.10/3.70 and 4.57/5.09 ppm arise from H_3_/H_4_ and H_2_/H_3_, respectively. Based on the chemical shifts of protons of CA, the strong correlations at δ_C_/δ_H_ 99.79/4.69, 71.62/4.54, 72.61/5.09, 76.59/3.68, 72.03/3.84, 62.49/4.01, 62.54/4.24 ppm in the HSQC spectrum of CA with DS of 2.75 (Figure S2B) are attributed to C_1_/H_1_, C_2_/H_2_, C_3_/H_3_, C_4_/H_4_, C_5_/H_5_, C_6`_/H_6`_ and C_6_/H_6_, while the strong correlations at δ_C_/δ_H_ 21.02/2.08, 20.67/1.95, 20.59/1.88 ppm are characteristic of the correlations of methyl group, thus further confirming the successful transesterification of cellulose in AmimCl/DMSO system. 

**Figure 2:**
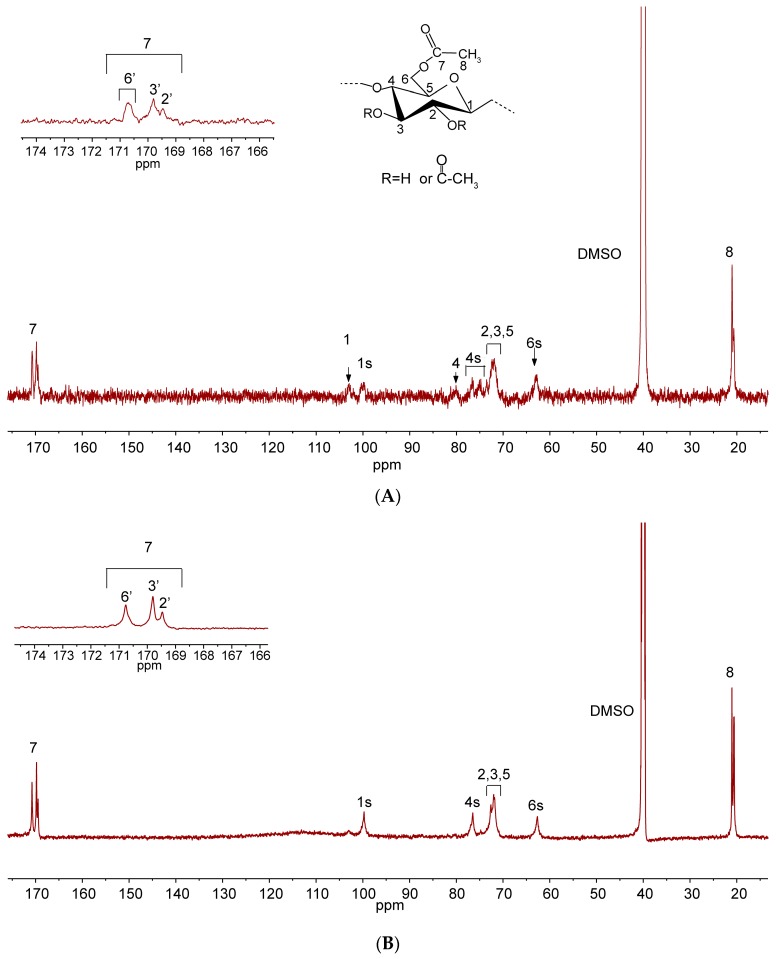
^13^C-NMR spectra of CA-3 (DS = 2.18, **A**) and CA-17 (DS = 2.75, **B**).

Figure 2 illustrates the difference between cellulose triacetate (DS = 2.75) and partially acetylated cellulose (DS = 2.18) reflected in ^13^C-NMR spectra. The chemical shifts of the partially acetylated cellulose were as follows: signals at 103.03, 99.88, 80.05, 76.63, 71.62–72.45, and 62.67 ppm are assigned to C_1_, C_1S_ (the hydroxyl group of C_2_ is substituted), C_4_, C_4S_ (the hydroxyl group of C_3_ is substituted), integrated carbons (C_2_, C_3_ and C_5_), and C_6S_ (the hydroxyl group of C-6 is substituted) of the AGU, respectively. The signals at 170.74, 169.80, 169.47 ppm correspond to carbonyl carbons at the position 7 and the signals at 20.65 and 21.03 ppm are associated with the methyl carbon at the position 8, providing direct evidence of the occurrence of the DBU-catalyzed transesterification in the cosolvent system. Compared with spectrum A, the absences of the signals of C_1_ and C_4_ in spectrum B confirmed that almost all the hydroxyl groups in the AGU were reacted with acyl groups. Moreover, the inset in the upper left corner shows three clearly resolved carbon resonances, indicating the structure of cellulose triacetate. The percentages of substitution of hydroxyl groups at different positions could be calculated by integrating the unmodified and esterified signals for C_2_, C_3_, and C_6_ of AGU from ^13^C-NMR and HSQC spectra [25]. In the present study, the percentages of substitution of C_2_-OH, C_3_-OH, and C_6_-OH of sample CA-17 were 50.6%, 49.1%, and 100%, respectively, suggesting the reactivity of hydroxyl groups was as follows: C_6_-OH > C_2_-OH > C_3_-OH. This was consistent with the results in the reported literature [22].

### 2.4. TGA/DTG Analysis

**Figure 3:**
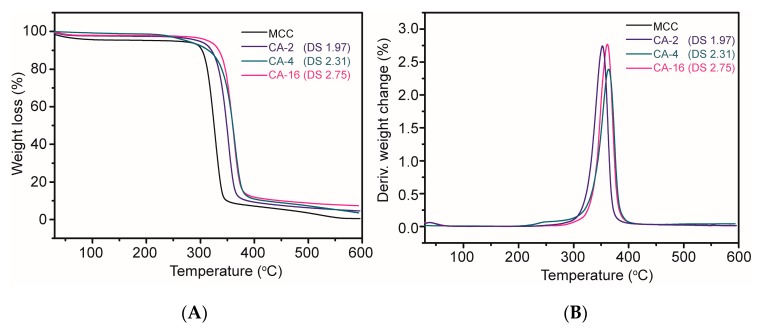
TGA (**A**) and DTG (**B**) spectra of MCC and modified samples.

In order to investigate the thermal properties of the raw material (MCC) and the synthesized CA samples, selected samples were characterized by TGA measurement in a N_2_ atmosphere. Figure 3A shows the TGA curves of MCC and the prepared CA samples with DS of 1.97, 2.31 and 2.75, respectively. From 30 °C to 100 °C, the weight of MCC was slightly lower due to the removal of water, while the loss of the CA samples were not obvious, revealing that the CA samples were less likely to absorb water than MCC [26]. MCC started to decompose at around 290 °C while the CA samples started to decompose at slightly higher temperature and yielded more pyrolysis residues. Figure 3B shows the DTG curves of the samples. The maximum degradation rate of the modified samples appeared at higher temperature than that of MCC, indicating that the thermal properties of the synthesized CA were slightly improved by introducing the acetyl group into cellulose. In addition, the thermal stability increased with increasing DS, in accordance with previous reports [27,28].

### 2.5. XRD

**Figure 4:**
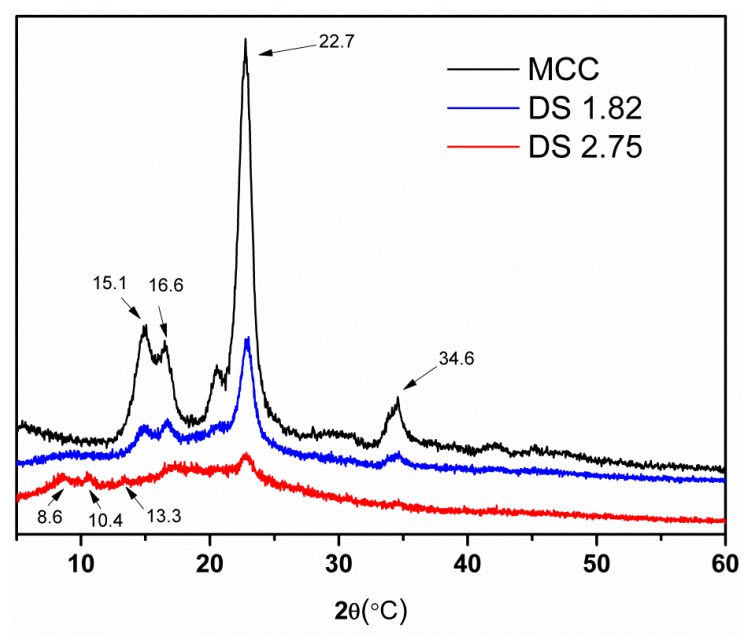
XRD patterns of MCC and CA samples with DS of 1.82 and 2.75.

The XRD patterns of MCC and the CA samples are shown in Figure 4. The diffraction peaks at 2θ = 15.1°, 16.6°, 22.7° and 34.6° are assigned to the (1 −1 0), (1 1 0), (2 0 0) and (0 0 4) diffraction planes, reflecting the cellulose I structure of the raw material MCC. It should be noted that, in the patterns of the CA samples, the intensity of these peaks decreased, and new peaks appeared at 2θ = 8.3°, 10.4° and 13.3° indicating changes in structure upon transesterification in the AmimCl/DMSO cosolvent system. Moreover, compared to MCC, the crystallinity of the CA samples gradually decreased with the increased DS, indicating a reduction in crystallinity.

### 2.6. Intrinsic Viscosity Analysis

The Mark-Houwink-Sakuruda equation (Equation (1)) describes the relationship between intrinsic viscosity and molecular weight of the cellulose derivatives.

(1) $$\left[\eta \right]=K{\left(\bar{M_{v}}\right)}^{a}$$

where *K* and *a* are constants dependent on the type of polymer and solvent at a certain temperature.

**Figure 5:**
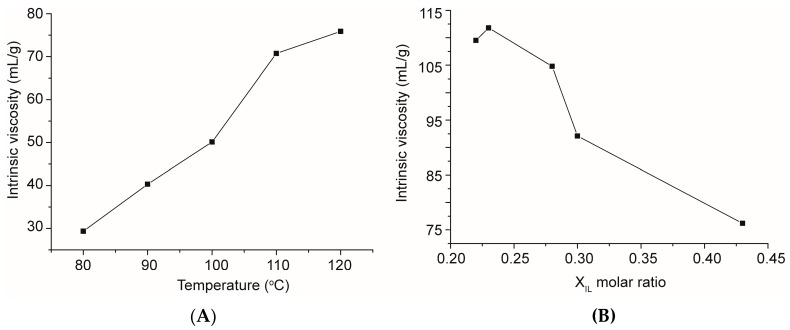
Intrinsic viscosity of cellulose acetates prepared at different temperatures (**A**) and with various molar ratios of IL (**B**).

Although *K* and *a* are not proposed for CA in DMSO, the changes of the molecular weight can be still inferred through the variation of the intrinsic viscosity of the CA solutions, because the intrinsic viscosity of cellulose derivatives depends on their molecular weight. Herein, sample CA 1~5 and CA 13~17 were used to investigate the effect of reaction temperature and molar ratio of IL: cosolvent on the intrinsic viscosity of the resulting CA. As it can be seen from Figure 5, there was a clear increase in the intrinsic viscosity when increasing the reaction temperature from 80 °C to 120 °C (A) and decreasing the molar ratio of IL from 0.43 to 0.22 (B). The result here was in accordance with the data in Table 1: the increase in DS increased the molecular weight of CA, which led to an increase in the intrinsic viscosity of the CA solution. No degradation was observed even at high temperature (120 °C) and at 6 h reaction time, suggesting that this is a promising method to produce CA via transesterification in AmimCl/DMSO cosolvent system, using DBU and IPA as super base catalyst and acetylation reagent, respectively.

## 3. Materials and Methods

### 3.1. Materials

MCC was purchased from Sinopharm Chemical Reagent Company (Shanghai, China). DMSO, IPA and DBU were purchased from Sigma-Aldrich Co. (Guangzhou, China). DMF was purchased from Jinhuada Chemical Reagent Co. Ltd. (Guangzhou, China). AmimCl with purity of 99% was purchased from Cheng-Jie Chemical Co., Ltd. (Shanghai, China) and used without further purification. Other chemicals (analytical grade) were purchased from Guangzhou Chemical Reagent Factory (Guangzhou, China) and used as received.

### 3.2. Synthesis of CA via Transesterification

MCC (0.2 g) was dispersed in 3 g of DMSO, or DMF with stirring. Then 10 g of AmimCl was added to the mixture and heated to a certain temperature (80–120 °C). After the cellulose was dissolved, 1.8 mL of IPA and 0.3 mL of DBU (corresponding to the molar ratio of IPA/AGU 13.3:1 and DBU/IPA 1:6) were added into the solution under agitation. After the required time, 200 mL of 99 wt % ethanol was poured into the solution. The precipitates were centrifuged, washed thoroughly with ethanol (three times, total 600 mL) and dried in the vacuum oven at 50 °C for 24 h.

### 3.3. Characterization

#### 3.3.1. FT-IR

Samples and KBr was dried in the oven at 105 °C for 8 h. The samples and KBr were mixed together at a ratio of 2:98 (*w*/*w*), ground, and pressed into a disc. The FT-IR spectra of samples were recorded using the disk on a Bruker spectrophotometer (Bruker, Karlsruhe, Germany) in the range of 400–4000 cm^−1^ with a resolution of 4 cm^−1^.

#### 3.3.2. XRD

X-ray diffraction of samples were performed on a D8 Advance instrument (Bruker AXS, Karlsruhe, Germany) with Nickel-filtered Cu Kα radiation (wavelength = 0.154 nm). The detailed data were as follows: diffraction angle 2θ, 5 to 60°; step size, 0.04°; and time per step, 0.2 s.

#### 3.3.3. TGA/DTG

The thermal stability of samples was characterized by using TGA and DTG on a thermogravimetric analyzer TA Q500 (TA Instruments, New Castle, DE, USA). The device was flushed with nitrogen continually. The samples weighed about 9–11 mg, and were heated from 30 to 600 °C at a heating rate of 10 °C/min.

#### 3.3.4. NMR Analysis

The ^1^H-NMR, ^13^C-NMR, ^1^H-^1^H COSY, and ^1^H-^13^C HSQC spectra of the CA samples were recorded from 40 mg in 0.5 mL of DMSO-*d*_6_ on a Bruker Advance III 600 M (Bruker, Karlsruhe, Germany) with a 5 mm multinuclear probe. For ^1^H-NMR analysis, the detailed collecting and processing parameters were as follows: number of scans, 16; receiver gain, 31; acquisition time, 2.7263 s; relaxation delay, 1.0 s; pulse width, 11.0 s; spectrometer frequency, 600.17 MHz; and spectral width, 12,019.2 Hz. For ^13^C-NMR, the detailed collecting and processing parameters were as follows: number of scans, 10,000; receiver gain, 31; acquisition time, 0.9088 s; relaxation delay, 2.0 s; pulse width, 12.0 s; spectrometer frequency, 150.91 MHz; and spectral width, 36,057.7 Hz. For ^1^H-^1^H COSY analysis, the detailed collecting and processing parameters were as follows: number of scans, 32; receiver gain, 18; acquisition time, 0.1024 s; relaxation delay, 1.5254 s; pulse width, 8.3 s; spectrometer frequency, 600.17/600.17 MHz; and spectral width, 10,000/10,000 Hz. For HSQC analysis, the detailed collecting and processing parameters were as follows: number of scans, 88; receiver gain, 187; acquisition time, 0.1420 s; relaxation delay, 1.0 s; pulse width, 12.3 s; spectrometer frequency, 600.17/150.91 MHz; and spectral width, 7211.5/24,875.6 Hz.

#### 3.3.5. Determination of DS

The DS of CA was calculated with the peak intensity of the corresponding resonances from the ^1^H-NMR spectra through the Equation (2) as reported previously [29,30,31]:

(2) $$\mathrm{DS}=7\times \frac{I_{\left(CH_{3},H\right)}}{\left[3\times I_{\left(AGU,H\right)}\right]}$$

where $I_{\left(CH_{3},H\right)}$ is the integrated area of the resonances assigned to methyl protons of the acetyl group, 7 and 3 are the numbers of protons in the glucose ring and methyl group, respectively, and $I_{\left(AGU,H\right)}$ is the integrated area of the resonances assigned to protons of the glucose ring.

#### 3.3.6. Intrinsic Viscosity Analysis

The CA samples were dissolved in DMSO at a concentration of 2 mg/L. The intrinsic viscosity was measured by Ubbelohde viscometer (Shanghai Shendi Glass Instrument Co. Ltd, Shanghai, China) at 25 °C. Using the single-point method [32], the intrinsic viscosity was calculated by the following equations:

(3) $$[\eta ]=\frac{\sqrt{2(\eta_{sp}-\ln\left(\eta_{r}\right))}}{C}$$

(4) $$\eta_{sp}=\eta_{r}-1$$

(5) $$\eta_{r}=t/t_{0}$$

where [*η*] is the intrinsic viscosity, *η_r_* is the relative viscosity, *η_sp_* is the specific viscosity, *C* is the concentration of the solution, and *t* and *t_0_* are the solution and solvent efflux times, respectively.

## 4. Conclusions

CA was successfully synthesized by transesterification with DBU and IPA as super base catalyst and acylation reagent, respectively, in AmimCl/cosolvent system. DMSO proved to be a more effective cosolvent in this reaction than DMF, under the conditions tested. The DS of the CA samples could be modulated from 1.82 to 2.75 by changing the ratio of DMSO to IL and the reaction temperature. The chemical structure of the CA samples was confirmed by FT-IR, XRD, and NMR analysis. The intrinsic viscosity of the CA samples increased with the growth of DS, while TGA/DTG analysis showed that the thermal stability of the CA samples was improved. The results suggested that DMSO was a suitable cosolvent for the transesterification, which provides a promising method to produce CA in this homogeneous system.

## Figures and Tables

**Table 1:** Conditions of cellulose acetylation in different cosolvents and the solubility of the obtained CA.

Entry	T (°C)	Time (h)	Solvent ^a^	DS	Solubility ^b^		
					DMSO	DMF	Acetone
CA-1	80	3	IL_0.6_/DMSO_0.4_	1.82	++	+	−
CA-2	90	3	IL_0.6_/DMSO_0.4_	1.97	++	+	−
CA-3	100	3	IL_0.6_/DMSO_0.4_	2.16	++	+	−
CA-4	110	3	IL_0.6_/DMSO_0.4_	2.31	++	+	++
CA-5	120	3	IL_0.6_/DMSO_0.4_	2.38	++	+	++
CA-6	100	3	IL_0.6_/DMF_0.4_	1.82	++	+	−
CA-7	110	3	IL_0.6_/DMF_0.4_	2.01	++	+	+
CA-8	120	3	IL_0.6_/DMF_0.4_	2.06	++	+	+
CA-9	100	3	IL	1.84	++	+	−
CA-10	110	3	IL	2.24	++	+	++
CA-11	120	3	IL	2.35	++	+	++
CA-12	120	6	IL	2.36	++	+	++
CA-13	120	6	IL_0.43_/DMSO_0.57_	2.38	++	+	++
CA-14	120	6	IL_0.3_/DMSO_0.7_	2.51	++	+	++
CA-15	120	6	IL_0.28_/DMSO_0.72_	2.59	++	+	++
CA-16	120	6	IL_0.23_/DMSO_0.77_	2.75	++	+	++
CA-17	120	6	IL_0.22_/DMSO_0.78_	2.71	++	+	++

**^a^** The molar ratio of IL to cosolvent; ^b^ Soluble (++), swollen (+) and insoluble (−) in the solvent.

## Supplementary Materials

The following are available online. Figure S1: ^1^H-NMR spectra of CA (DS = 2.75), Figure S2: ^1^H-^1^H COSY (**A**) and HSQC spectra (**B**) of the CA sample with DS of 2.75.

## References

[1] Heinze T., Liebert T. (2004). 4.2 Chemical characteristics of cellulose acetate. Macromol. Symp..

[2] Bilal M., Asgher M., Iqbal H.M., Hu H., Zhang X. (2017). Biotransformation of lignocellulosic materials into value-added products—A review. Int. J. Biol. Macromol..

[3] Tang Z., Fu Y., Ma Z. (2017). Bovine serum albumin as an effective sensitivity enhancer for peptide-based amperometric biosensor for ultrasensitive detection of prostate specific antigen. Biosens. Bioelectron..

[4] Longsheng Z., Wei F., Tianxi L. (2016). Flexible hierarchical membranes of WS 2 nanosheets grown on graphene-wrapped electrospun carbon nanofibers as advanced anodes for highly reversible lithium storage. Nanoscale.

[5] Cai J., Zhang L. (2005). Rapid dissolution of cellulose in LiOH/UREA and NaOH/UREA aqueous solutions. Macromol. Biosci..

[6] Vitz J., Erdmenger T., Haensch C., Schubert U.S. (2009). Extended dissolution studies of cellulose in imidazolium based ionic liquids. Green Chem..

[7] Schlufter K., Schmauder H.-P., Dorn S., Heinze T. (2006). Efficient homogeneous chemical modification of bacterial cellulose in the ionic liquid 1-*N*-butyl-3-methylimidazolium chloride. Macromol. Rapid Commun..

[8] Wu J., Zhang J., Zhang H., He J.S., Ren Q., Guo M. (2004). Homogeneous acetylation of cellulose in a new ionic liquid. Biomacromolecules.

[9] Pinkert A., Marsh K.N., Pang S., Staiger M.P. (2009). Ionic liquids and their interaction with cellulose. Chem. Rev..

[10] Chen J., Chen X., Su M., Ye J., Hong J., Yang Z. (2015). Direct production of all-wood plastics by kneading in ionic liquids/DMSO. Chem. Eng. J..

[11] Gu S., Wang J., Wei X., Cui H., Wu X., Wu F. (2014). Enhancement of lipase-catalyzed synthesis of caffeic acid phenethyl ester in ionic liquid with dmso co-solvent. Chin. J. Chem. Eng..

[12] Jogunola O., Eta V., Hedenstrom M., Sundman O., Salmi T., Mikkola J.P. (2016). Ionic liquid mediated technology for synthesis of cellulose acetates using different co-solvents. Carbohydr. Polym..

[13] Rinaldi R. (2011). Instantaneous dissolution of cellulose in organic electrolyte solutions. Chem. Commun. (Camb.).

[14] Gale E., Wirawan R.H., Silveira R.L., Pereira C.S., Johns M.A., Skaf M.S., Scott J.L. (2016). Directed discovery of greener cosolvents: New cosolvents for use in ionic liquid based organic electrolyte solutions for cellulose dissolution. ACS Sustain. Chem. Eng..

[15] Kakuchi R., Ito R., Nomura S., Abroshan H., Ninomiya K., Ikai T., Maeda K., Kim H.J., Takahashi K. (2017). A mechanistic insight into the organocatalytic properties of imidazolium-based ionic liquids and a positive co-solvent effect on cellulose modification reactions in an ionic liquid. RSC Adv..

[16] Cao Y., Wu J., Zhang J., Li H., Zhang Y., He J. (2009). Room temperature ionic liquids (RTILS): A new and versatile platform for cellulose processing and derivatization. Chem. Eng. J..

[17] Kilpeläinen I., Xie H., King A., Granstrom M., Heikkinen S., Argyropoulos D.S. (2007). Dissolution of wood in ionic liquids. J. Agric. Food Chem..

[18] Schenzel A., Hufendiek A., Barner-Kowollik C., Meier M.A.R. (2014). Catalytic transesterification of cellulose in ionic liquids: Sustainable access to cellulose esters. Green Chem..

[19] Sung Jun L., Hae Sung L., Sang Won J., Hyun-Chul K., Se Geun L., Tae Hwan O. (2015). Effect of dimethyl sulfoxide on synthesis of thermoplastic cellulose-graft-poly(l-lactide) copolymer using ionic liquid as reaction media. J. Appl. Polym. Sci..

[20] Casarano R., El Seoud O.A. (2013). Successful application of an ionic liquid carrying the fluoride counter-ion in biomacromolecular chemistry: Microwave-assisted acylation of cellulose in the presence of 1-allyl-3-methylimidazolium fluoride/DMSO mixtures. Macromol. Biosci..

[21] Cao X., Sun S., Peng X., Zhong L., Sun R., Jiang D. (2013). Rapid synthesis of cellulose esters by transesterification of cellulose with vinyl esters under the catalysis of NaOH or KOH in DMSO. J. Agric. Food Chem..

[22] Achtel C., Heinze T. (2016). Homogeneous acetylation of cellulose in the new solvent triethyloctylammonium chloride in combination with organic liquids. Macromol. Chem. Phys..

[23] Celebioglu A., Demirci S., Uyar T. (2014). Cyclodextrin-grafted electrospun cellulose acetate nanofibers via “click” reaction for removal of phenanthrene. Appl. Surf. Sci..

[24] Kono H., Hashimoto H., Shimizu Y. (2015). NMR characterization of cellulose acetate: Chemical shift assignments, substituent effects, and chemical shift additivity. Carbohydr. Polym..

[25] Wang H., Chen Y., Wei Y., Zhang A., Liu C. (2017). Homogeneous esterification mechanism of bagasse modified with phthalic anhydride in ionic liquid, Part 2: Reactive behavior of hemicelluloses. Carbohydr. Polym..

[26] Chen J., Xu J., Wang K., Cao X., Sun R. (2016). Cellulose acetate fibers prepared from different raw materials with rapid synthesis method. Carbohydr. Polym..

[27] Barud H.S., de Araujo Junior A.M., Santos D.B., de Assuncao R.M.N., Meireles C.S., Cerqueira D.A., Rodrigues Filho G., Ribeiro C.A., Messaddeq Y., Ribeiro S.J.L. (2008). Thermal behavior of cellulose acetate produced from homogeneous acetylation of bacterial cellulose. Thermochim. Acta.

[28] Tosh B. (2011). Thermal analysis of cellulose esters prepared from different molecular weight fractions of high alpha-cellulose pulp. Indian J. Chem. Technol..

[29] Ass B.A., Ciacco G.T., Frollini E. (2006). Cellulose acetates from linters and sisal: Correlation between synthesis conditions in DMAC/LiCL and product properties. Bioresour. Technol..

[30] Elomaa M. (2004). Determination of the degree of substitution of acetylated starch by hydrolysis, ^1^H NMR and TGA/IR. Carbohydr. Polym..

[31] Sun X., Lu C., Zhang W., Tian D., Zhang X. (2013). Acetone-soluble cellulose acetate extracted from waste blended fabrics via ionic liquid catalyzed acetylation. Carbohydr. Polym..

[32] Pamies R., Hernandez Cifre J.G., Lopez Martinez M.D.C., Garcia de la Torre J. (2008). Determination of intrinsic viscosities of macromolecules and nanoparticles, Comparison of single-point and dilution procedures. Colloid Polym. Sci..

