# Supplementary material for: Acetylation of Microcrystalline Cellulose by Transesterification in AmimCl/DMSO Cosolvent System
Source: Molecules. 2017 Aug 27;22(9):1419. doi: 10.3390/molecules22091419 (PMC6151486; doi:10.3390/molecules22091419)
Supplement: Supplementary file 1 [file molecules-22-01419-s001.pdf]

Acetylation of Microcrystalline Cellulose by Transesterification in AmimCl/DMSO Cosolvent System

Huihui Wang [1], Xiaoxiang Wen [1], Xueqin Zhang [1], and Chuanfu Liu [1,*]

[1] State Key Laboratory of Pulp and Paper Engineering, South China University of Technology, Guangzhou 510640, China; wang.huihui@mail.scut.edu.cn (H.W.); 15902078732@163.com (X.W.); xueqin0228@gmail.com (X.Z.)

* Correspondence: chfliu@scut.edu.cn; Tel.: +86-20-87111735

Figure Captions

Figure S1. ^{1}H-NMR spectra of CA (DS=2.75).

Figure S2. ^{1}H-^{1}H COSY (A) and HSQC spectra (B) of the CA sample with DS of 2.75.

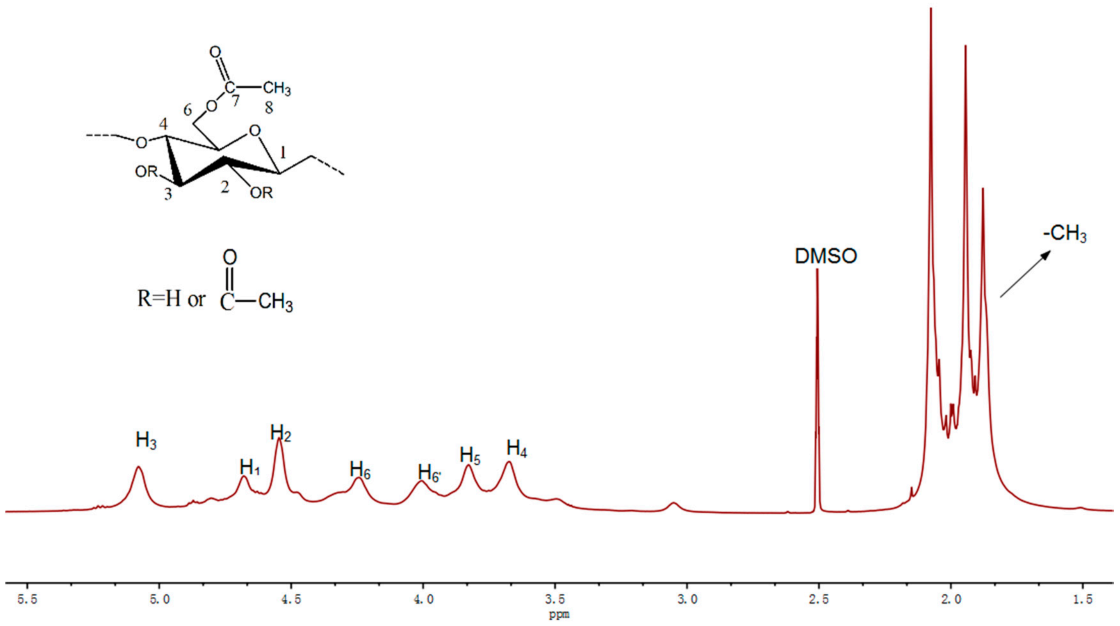

Figure S1. ^{1}H-NMR spectra of CA (DS=2.75).

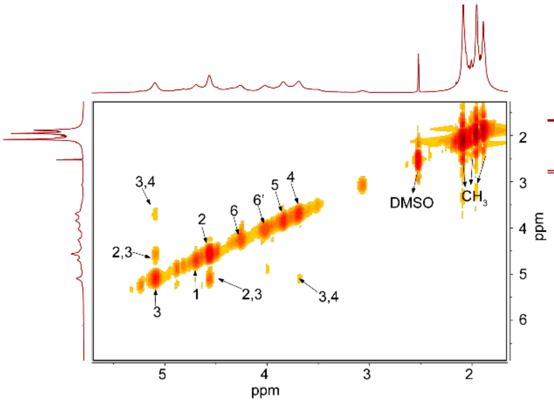

(**A**)

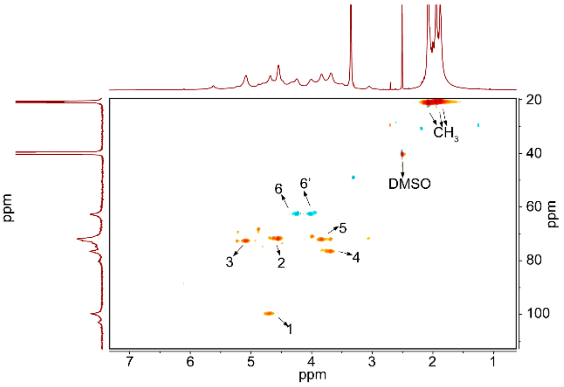

(**B**)

Figure S2. ^1H-^1H COSY (A) and HSQC spectra (B) of the CA sample with DS of 2.75.
